# Does daily co administration of gonadotropins and letrozole during the ovarian stimulation improve IVF outcome for poor and sub optimal responders?

**DOI:** 10.1186/s13048-020-00666-z

**Published:** 2020-06-08

**Authors:** Moran Shapira, Raoul Orvieto, Oshrit Lebovitz, Ravit Nahum, Adva Aizer, Aliza Segev-Zahav, Jigal Haas

**Affiliations:** IVF unit, Division of Obstetrics and Gynecology, Sheba Medical Center, Sackler School of Medicine, Tel-Aviv University, Tel-Aviv, Israel

**Keywords:** Letrozole, Aromatase inhibitors, Poor responders, Androgens, IVF

## Abstract

**Background:**

Co-administration of letrozole during the first 5 days of ovarian stimulation was suggested to improve IVF outcomes in poor responders. We aimed to determine whether poor/sub-optimal responders might benefit from Letrozole co-treatment throughout the entire stimulation course.

**Methods:**

We retrospectively reviewed the medical files of women who demonstrated poor (oocyte yield ≤3) and sub-optimal (4 ≤ oocyte yield ≤9) ovarian response during conventional multiple-dose antagonist stimulation protocols and were co-treated in a subsequent cycle with 5 mg Letrozole from the first day of stimulation until trigger day. A self-paired comparison between gonadotropins-only and gonadotropins-letrozole cycles was performed.

**Results:**

Twenty-four patients were included. Mean patients’ age was 39.83 ± 4.60 and mean day-3-FSH was 12.77 ± 4.49 IU/m. Duration of stimulation and total gonadotropins dose were comparable between the two cycle groups. Peak estradiol levels were significantly lower in gonadotropins-letrozole cycles (2786.74 ± 2118.53 vs 1200.13 ± 535.98, *p* < 0.05). Number of retrieved oocytes (3.29 ± 2.15 vs 6.46 ± 3.20, *p* < 0.05), MII-oocytes (2.47 ± 1.65 vs 5.59 ± 3.20, *p* < 0.05), 2PN-embryos (1.78 ± 1.50, 4.04 ± 2.74, *p* < 0.05) and top-quality embryos (0.91 ± 0.97 vs. 2.35 ± 1.66, *p* < 0.05) were significantly higher in the gonadotropins-letrozole cycles. Clinical pregnancy rate in gonadotropins-letrozole cycles was 31.5%.

**Conclusion:**

Letrozole co-treatment during the entire stimulation course improves ovarian response and IVF outcomes in poor/sub-optimal responders.

## Background

The role of androgens in female reproduction has been extensively studied in the past two decades. It is now clear that androgens, although traditionally thought to be male sex steroids, are significantly involved in regulating normal and pathological female reproductive states. Androgens serve as a substrate for estradiol production, promote the proliferation of granulosa and theca cells, stimulate the growth of small follicles and increase FSH receptor gene expression as well as IGF-I and IGF-I receptors [1–3] . This knowledge has prompted the incorporation of androgens and androgen-modulating agents into the clinical practice of assisted reproduction technologies (ART). One such androgen modulating agent is Letrozole, a selective, non-steroidal aromatase inhibitor. It competitively binds to the heme of the cytochrome P450 subunit of the aromatase enzyme, thereby blocking the conversion of androstenedione and testosterone to estrone and estradiol, respectively.

Several studies evaluated the co-administration of Letrozole during ovarian stimulation in patients suffering from diminished ovarian reserve, yielding conflicting results [4–6] . In these studies, Letrozole was started either concomitantly with or prior to gonadotropins, and was given for a total of 5 stimulation days. Hypothetically, extending the duration of letrozole co-treatment may provide a more pronounced effect on intrafollicular androgens and circulating estrogen levels. We have previously reported that Letrozole co-treatment during the entire stimulation course improves ovarian response in normal responders undergoing IVF-ET [7]. In this study we aimed to determine the value of Letrozole co-treatment throughout the entire stimulation course in poor and sub-optimal responders.

## Methods

We retrospectively reviewed the medical files of women who were treated in our IVF unit over a one-year period and demonstrated poor or sub-optimal ovarian response during a standard multiple-dose GnRH antagonist IVF stimulation cycle. Poor and sub-optimal ovarian responses were defined as oocyte yields of 1–3 and 4–9 per cycle, respectively [8]. Included in the study were only those patients whose subsequent IVF cycle attempt involved co-administration of 5 mg Letrozole from the first day of gonadotropin stimulation until trigger day (gonadotropins-letrozole cycle). Only fresh IVF stimulation cycles involving the GnRH antagonist protocol were included. Patients were excluded whenever the time interval between the two cycles of interest exceeded 6 months. Demographic data and infertility treatment related variables were collected from the files. Gonadotropins-only cycles and gonadotropins-letrozole cycles were compared with regard to ovarian stimulation characteristics and IVF outcomes. Institutional review board approval was obtained.

Ovarian stimulation was initiated on day 2–3 of menstrual cycle, using recombinant FSH)Gonal-F, Merck-Serono(. The starting dose of gonadotropins ranged from 300 to 450 IU and was determined according to patient’s age, body mass index (BMI), hormone profile, antral follicular count and previous response to stimulation. In letrozole-gonadotropins cycle, letrozole (FEMARA®, Novartis Pharma Stain AG**,** Switzerland) was added from the first day of stimulation. Once the leading follicle had reached 13 mm and/or serum estradiol level was ≥1200 pmol/L, a daily dose of 0.25 mg GnRH antagonist (Cetrotide®, Merck Serono, Darmstadt, Germany) was initiated and recombinant FSH was substituted by gonadotropin preparations containing LH-activity (MENOPUR- Ferring Pharmaceuticals, Copenhagen, Denmark; or, PERGOVERIS, Merck Serono, Darmstadt, Germany). Gonadotropins doses were adjusted throughout the cycle based on ultrasound monitoring and estradiol levels. Triggering for final oocyte maturation was performed when the leading follicle reached 17 mm. Transvaginal oocyte retrieval was performed 36 h following trigger. Classification of embryo quality was based on previously published scoring parameters [9]; a top-quality embryo was defined as four to five blastomeres on day 2, seven or more blastomeres on day 3, equally-sized blastomeres and ≤ 15% fragmentation on day 3 and no multinucleation. Luteal support was initiated 1 day after oocyte pick-up and consisted of Vaginal progesterone gel 90 mg/day 8% (CRINONE; Serono). Following a positive pregnancy test, ongoing pregnancies were confirmed by presence of gestational sac with fetal heart rate on ultrasound in 6–8 weeks gestation.

Statistical analysis was conducted using SciPy, version 1.0.0. Continuous variables were presented as means and standard deviation (SD) or medians and IQR, as appropriate. Categorical variables were presented as numbers and percentages. Normally distributed continuous variables were compared using Student’s paired t-test, and non-normally distributed numbers were compared using the Mann-Whitney test. Categorical variables were compared using the chi-squared or Fisher’s exact test, as appropriate. A *p*-value < 0.05 was considered statistically significant.

## Results

Twenty-four patients met the inclusion criteria and were included in the study. Of these patients, fourteen had demonstrated poor response (oocytes< 4) in the first cycle included, whereas the remaining ten had demonstrated sub-optimal response (3 < oocyte< 10). Mean patients’ age was 39.83 ± 4.60and mean FSH on day 3 of cycle (FSHd3) was 12.77 ± 4.49 IU/L. Comparison between gonadotropins-only cycles and letrozole cycle is presented on Table 1. Total dose of gonadotropins (4744.79 ± 2109.60 vs 4820.83 ± 1620.50, *p* = 0.84) and length of stimulation (10.82 ± 3.17 vs 10.50 ± 2.57, 0 = 0.72) were comparable between the two cycle groups. Estradiol levels were significantly lower in gonadotropin-letrozole cycles (2786.74 ± 2118.53 vs 1200.13 ± 535.98, *p* < 0.05). Numbers of follicles > 11 mm (4.00 ± 2.65 vs 5.96 ± 2.68, p < 0.05) and > 16 mm at trigger day (2.61 ± 1.44 vs 4.04 ± 1.83, *p* = 0.05) were significantly higher in gonadotropin-letrozole cycles. A median increase of 200% (IQR 125–300%) in oocyte yield was observed, leading to a significantly higher number of retrieved oocytes in letrozole cycles (3.29 ± 2.15 vs 6.46 ± 3.20, *p* < 0.05). MII oocytes (2.47 ± 1.65 vs 5.59 ± 3.20, *p* < 0.05), 2PN embryos (1.78 ± 1.50, 4.04 ± 2.74, *p* < 0.05) and quality embryos (0.91 ± 0.97 vs. 2.35 ± 1.66, *p* < 0.05) were significantly higher in gonadotropin-letrozolecycles as well. A total of 6 pregnancies were achieved following gonadotropin-letrozole cycles, one of which was ectopic, and another resulted in spontaneous early abortion. Pregnancy rates could be calculated for 19 of the 24 participating patients (2 patients had yet to undergo embryo transfer, 2 patients stored their embryos, and another patient performed prenatal genetic diagnosis revealing abnormal embryos). Thus, a clinical pregnancy rate of 31.5% and an ongoing pregnancy rate of 21% was observed. A sub-group analysis according to initial ovarian response is presented on Table 2. In poor responders, all IVF outcome measures were significantly improved in gonadotropin-letrozole cycles. As for sub-optimal responders, number of follicles > 11 mm and > 16 mm at trigger day, as well as number of M2 oocytes, were significantly higher in gonadotropin-letrozole cycles. The remaining outcome measures were also improved in gonadotropin-letrozole cycles, but the differences did not reach statistical significance.

**Table 1:** IVF outcomes of gonadotropins only and letrozole- gonadotropins cycles

	Poor + sub-optimal responders (*N* = 24)		
	Letrozole (−)	Letrozole (+)	p
Stimulation days	10.82 ± 3.17	10.50 ± 2.57	0.72
Total FSH (IU/mL)	4744 ± 2109	4820 ± 1620	0.85
Max E2 (pmol/L)	2786 ± 2118	1200 ± 536	< 0.05
Follicles> 11 mm	4.00 ± 2.65	5.96 ± 2.68	< 0.05
Follicles≥16 mm	2.61 ± 1.44	4.04 ± 1.83	< 0.05
OPU	3.29 ± 2.15	6.46 ± 3.20	< 0.05
M2 oocytes	2.47 ± 1.65	5.59 ± 3.20	< 0.05
Fertilization rate	0.57 ± 0.36	0.66 ± 0.24	0.37
2PN embryos	1.78 ± 1.50	4.04 ± 2.74	< 0.05
Top quality embryos	0.91 ± 0.97	2.35 ± 1.66	< 0.05

**Table 2:** IVF outcomes in poor and suboptimal responders

	Poor responders (*N* = 14)			Sub-optimal responders (*N* = 10)		
	Letrozole (−)	Letrozole (+)	P	Letrozole (−)	Letrozole (+)	p
Age	40.36 ± 4.01			39.1 ± 5.22		
BMI	25.31 ± 5.79			26.44 ± 6.22		
FSHd3	16.54 ± 3.07			9.47 ± 2.51		
Stimulation days	10.5 ± 3.12	10 ± 2.65	0.72	11.2 ± 3.19	11.10 ± 2.34	0.93
Total FSH	4714 ± 2207	4830 ± 1471	0.79	4787 ± 1962	4765 ± 1807	0.97
Max E2	2013 ± 1496	1160 ± 527	< 0.05	3675 ± 2452	1251 ± 543	< 0.05
Follicles> 11 mm	2.69 ± 1.77	4.54 ± 1.55	< 0.05	5.70 ± 2.65	7.80 ± 2.71	< 0.05
Follicles≥16 mm	1.92 ± 1.14	3.15 ± 1.35	< 0.05	3.50 ± 1.28	5.20 ± 1.72	< 0.05
OPU	1.79 ± 0.86	6 ± 2.78	< 0.05	5.40 ± 1.56	7.1 ± 3.62	0.14
M2 oocytes	1.5 ± 0.81	4.9 ± 3.18	< 0.05	3.22 ± 1.23	4.11 ± 2.18	< 0.05
Fertilization rate	0.51 ± 0.41	0.65 ± 0.26	0.35	0.67 ± 0.26	0.67 ± 0.2	0.96
2PN embryos	0.86 ± 0.74	4 ± 3.05	< 0.05	3.22 ± 1.23	4.11 ± 2.18	0.33
Top quality embryos	0.57 ± 0.62	2.57 ± 1.99	< 0.05	1.34 ± 1.17	2.00 ± 0.82	0.21

## Discussion

In the current study, we report the IVF outcomes of poor and suboptimal responders, undergoing IVF using letrozole co-administration for the entire ovarian stimulation period. Letrozole co-treatment resulted in improved outcomes, as demonstrated by increased oocytes retrieved, number of M2 oocytes, 2PN embryos and top-quality embryos.

Letrozole was first introduced into the ART practice for the purpose of ovulation induction, as an alternative to clomiphene citrate [10]. Shortly after, combined administration of Letrozole and gonadotropins was found to lower the required dose of gonadotropins, while maintaining or increasing the number of mature follicles observed during ovarian stimulation for intra uterine insemination (IUI) cycles [11, 12]. These observations prompted letrozole co-treatment in poor responder patients undergoing IVF cycles, aiming to improve ovarian response. Goswami et al. were the first to demonstrate the potential benefit of letrozole co-treatment in poor-responders undergoing IVF. In their study, patients receiving 2.5 mg letrozole for the first 5 days of stimulation with a low dose of r-FSH, had similar outcomes compared to patients treated with standard long GnRH-agonist protocol [4]. A study by Gracia-Velasco et al. has further demonstrated the value of co-treatment with letrozole. Co-administration of 2.5 mg letrozole for the first 5 days of ovarian stimulation during a high dose FSH/hMG GnRH-antagonist protocol, resulted in a higher number of retrieved oocytes and an increased implantation rate [5]. These encouraging findings, however, were not reproduced by a later, double-blinded randomized control study, where letrozole was added to a standard GnRH-antagonist protocol, at a dose and timing similar to the previous study presented [6]. Additional retrospective studies demonstrated that co-treatment with 5 mg letrozole resulted in reduction of gonadotropins dosage [13–15], while decreasing cancellation rates [13] and increasing pregnancy and live birth rates [14]. More recently, a double-blind randomized trial found an increase in retrieved and M2 oocytes among patients who were co-treated with 5 mg letrozole during the first 5 days of stimulation of rFSH/HMG antagonist protocol [16].

The beneficial effect of letrozole in the context of treating patients with compromised ovarian response can be explained by several mechanisms. Letrozole inhibits overall estrogens production, leading to withdrawal of negative feedback and a resultant increase in endogenous FSH production during the first days of cycle. Perhaps more importantly, letrozole increases intraovarian androgens, which are known to increase the expression of FSH receptors augment follicular sensitivity to FSH stimulation [17] and stimulate expression of insulin-like growth factor-1 which may act in synergy with FSH [3]. This mode of action may be particularly relevant for patients in their late 30’s or early 40’s, in restoring androgenic ovarian environment, which significantly changes as women age. Other than having a stimulatory role, it is possible that letrozole exerts its favorable impact through other modes of action; as supra-physiologic estradiol levels in ART cycles have been shown negatively affect oocyte quality and embryo implantation [18]. Thus, reduced serum E_2_ concentration achieved with letrozole may promote oocyte maturation and endometrial receptivity [19]. A study comparing between co-treatment with clomiphene citrate versus letrozole can further elucidate letrozole’s mode of action, by differentiating between the straightforward stimulatory effect on oocyte production shared by both agents, as opposed to the more intricate androgenic effect, which clomiphene lacks.

In view of the aforementioned proposed mechanisms, it is possible that extending letrozole administration to the entire stimulation course will promote follicular growth, oocyte quality and endometrial receptivity to a greater extent. In recent years, such protocol has been employed in breast cancer patients undergoing IVF for embryos/oocytes storage, with several studies showing an increase in oocyte yield and mature oocytes [20–22]. Other than for the purpose of fertility preservation, extended administration of letrozole has been evaluated in normal responders who were found to benefit from such treatment, as demonstrated by increase in oocyte yield, MII oocytes and blastocytes [7].

Poor responders represent a population of patients most likely to benefit from new treatment strategies, and the optimal stimulation protocol in this group of patients remains unknown [23]. In a recent meta-analysis, micro-dose GnRH agonist flare-up protocol was found superior to letrozole/antagonist protocol in terms of clinical pregnancy rates achieved by poor responders [24]. Nonetheless, to the best of our knowledge, only one study thus far has looked into extended letrozole administration in poor responders. The study compared the efficacy and cost-effectiveness of extended high dose letrozole/antagonist protocol (5 mg/day during the first 5 days of cycle and 2.5 mg/day during the subsequent 3 days) with short low dose letrozole/ antagonist protocol (2.5 mg letrozole for the first 5 days of cycle) [25]. Total gonadotropins dose and medications cost per cycle were significantly lower in extended letrozole group, while IVF outcomes were comparable in both protocols evaluated.

In the present study, letrozole was given during the entire ovarian stimulation course for an average of 10 days, using a fixed dose of 5 mg. Since each participant served as her own control, our data could reliably reflect the effect of letrozole co-treatment in patients suffering from low ovarian response, either poor or sub-optimalresponders. While using an equivalent cumulative dose of gonadotropins, a two-fold increase in ovarian response was observed following the gonadotropin-letrozole cycles, ultimately resulting in a mean oocyte yield of 6.5 and a total of 4 ongoing pregnancy pregnancies (ongoing pregnancy rate of 21%). It could be questioned whether the extended use of letrozole poses any teratogenic risk. An initial study found an increase in cardiac and musculoskeletal malformations in offspring of mothers who conceived with letrozole [26], however these findings were not substantiated by subsequent larger studies [27, 28].

A major strength of the current study is its self-controlled design. Self-paired comparison allows for a greater biological homogeneity, thereby reducing the impact of cofounding covariates. However, the self-paired design might have introduced a regression to the mean bias, which could hypothetically explain the improvement in cycle outcomes when co-treating with letrozole. Our study is also limited by its retrospective nature and small sample size. We suggest that further studies, preferably prospective, larger ones, should be performed in order to substantiate our findings.

## Conclusions

In conclusion, the herewith presented data demonstrated the efficacy of extended letrozole use during IVF cycles performed in patients with low ovarian response. Adding Letrozole during the whole ovarian stimulation course of rFSH/HMG GnRH-antagonist protocol significantly improved IVF outcomes for poor/sub-optimal responders and should be considered as a measure to increase conception rates in this subset of patients. Further studies, preferably randomized controlled trials, should validate the efficacy and safety of such protocol.

## Data Availability

Data will be made available from the corresponding author on request.

## References

[1] Gleicher N, Weghofer A, Barad DH (2011). The role of androgens in follicle maturation and ovulation induction: friend or foe of infertility treatment?. Reprod Biol Endocrinol.

[2] Lebbe M, Woodruff TK (2013). Involvement of androgens in ovarian health and disease. Mol Hum Reprod.

[3] Vendola K, Zhou J, Wang J, Bondy CA (1999). Androgens promote insulin-like growth factor-I and insulin-like growth factor-I receptor gene expression in the primate ovary. Hum Reprod.

[4] Goswami SK, Das T, Chattopadhyay R, Sawhney V, Kumar J (2004). A randomized single-blind controlled trial of letrozole as a low-cost IVF protocol in women with poor ovarian response: a preliminary report. Hum Reprod.

[5] Garcia-Velasco JA, Moreno L, Pacheco A, Guillen A, Duque L (2005). The aromatase inhibitor letrozole increases the concentration of intraovarian androgens and improves in vitro fertilization outcome in low responder patients: a pilot study. Fertil Steril.

[6] Ebrahimi M, Akbari-Asbagh F, Ghalandar-Attar M (2017). Letrozole+ GnRH antagonist stimulation protocol in poor ovarian responders undergoing intracytoplasmic sperm injection cycles: an RCT. Int J Reprod Biomed (Yazd).

[7] Haas J, Bassil R, Meriano J, Samara N, Barzilay E (2017). Does daily co-administration of letrozole and gonadotropins during ovarian stimulation improve IVF outcome?. Reprod Biol Endocrinol.

[8] Drakopoulos P, Blockeel C, Stoop D, Camus M, de Vos M (2016). Conventional ovarian stimulation and single embryo transfer for IVF/ICSI. How many oocytes do we need to maximize cumulative live birth rates after utilization of all fresh and frozen embryos?. Hum Reprod.

[9] Ziebe S, Lundin K, Janssens R, Helmgaard L, Arce JC, Group M (2007). Influence of ovarian stimulation with HP-hMG or recombinant FSH on embryo quality parameters in patients undergoing IVF. Hum Reprod.

[10] Mitwally MF, Casper RF (2001). Use of an aromatase inhibitor for induction of ovulation in patients with an inadequate response to clomiphene citrate. Fertil Steril.

[11] Mitwally MF, Casper RF (2002). Aromatase inhibition improves ovarian response to follicle-stimulating hormone in poor responders. Fertil Steril.

[12] Healey S, Tan SL, Tulandi T, Biljan MM (2003). Effects of letrozole on superovulation with gonadotropins in women undergoing intrauterine insemination. Fertil Steril.

[13] Ozmen B, Sonmezer M, Atabekoglu CS, Olmus H (2009). Use of aromatase inhibitors in poor-responder patients receiving GnRH antagonist protocols. Reprod BioMed Online.

[14] Lazer T, Dar S, Shlush E, Al Kudmani BS, Quach K (2014). Comparison of IVF outcomes between minimal stimulation and high-dose stimulation for patients with poor ovarian reserve. Int J Reprod Med.

[15] Bastu E, Buyru F, Ozsurmeli M, Demiral I, Dogan M, Yeh J (2016). A randomized, single-blind, prospective trial comparing three different gonadotropin doses with or without addition of letrozole during ovulation stimulation in patients with poor ovarian response. Eur J Obstet Gynecol Reprod Biol.

[16] Moini A, Lavasani Z, Kashani L, Mojtahedi MF, Yamini N (2019). Letrozole as co-treatment agent in ovarian stimulation antagonist protocol in poor responders: a double-blind randomized clinical trial. Int J Reprod Biomed (Yazd).

[17] Weil S, Vendola K, Zhou J, Bondy CA (1999). Androgen and follicle-stimulating hormone interactions in primate ovarian follicle development. J Clin Endocrinol Metab.

[18] Simon C, Cano F, Valbuena D, Remohi J, Pellicer A (1995). Clinical evidence for a detrimental effect on uterine receptivity of high serum oestradiol concentrations in high and normal responder patients. Hum Reprod.

[19] Mitwally MF, Casper RF, Diamond MP (2005). The role of aromatase inhibitors in ameliorating deleterious effects of ovarian stimulation on outcome of infertility treatment. Reprod Biol Endocrinol.

[20] Turan V, Bedoschi G, Emirdar V, Moy F, Oktay K (2018). Ovarian stimulation in patients with cancer: impact of Letrozole and BRCA mutations on fertility preservation cycle outcomes. Reprod Sci.

[21] Pereira N, Hancock K, Cordeiro CN, Lekovich JP, Schattman GL, Rosenwaks Z (2016). Comparison of ovarian stimulation response in patients with breast cancer undergoing ovarian stimulation with letrozole and gonadotropins to patients undergoing ovarian stimulation with gonadotropins alone for elective cryopreservation of oocytesdagger. Gynecol Endocrinol.

[22] Lee S, Oktay K (2012). Does higher starting dose of FSH stimulation with letrozole improve fertility preservation outcomes in women with breast cancer?. Fertil Steril.

[23] Ben-Rafael Z, Orvieto R, Feldberg D (1994). The poor-responder patient in an in vitro fertilization-embryo transfer (IVF-ET) program. Gynecol Endocrinol.

[24] Song Y, Li Z, Wu X, Wang X, Xiao J, Wang B (2014). Effectiveness of the antagonist/letrozole protocol for treating poor responders undergoing in vitro fertilization/intracytoplasmic sperm injection: a systematic review and meta-analysis. Gynecol Endocrinol.

[25] Fouda UM, Sayed AM (2011). Extended high dose letrozole regimen versus short low dose letrozole regimen as an adjuvant to gonadotropin releasing hormone antagonist protocol in poor responders undergoing IVF-ET. Gynecol Endocrinol.

[26] Biljan MHHR, Brassard N. The outcome of 150 babies following treatment with letrozole and gonadotropins. Fertil Steril. 2005;84(Suppl 1).

[27] Tulandi T, Martin J, Al-Fadhli R, Kabli N, Forman R, et al. Congenital malformations among 911 newborns conceived after infertility treatment with letrozole or clomiphene citrate. Fertil Steril. 2006;85:1761–5. 10.1016/j.fertnstert.2006.03.014 16650422

[28] Forman R, Gill S, Moretti M, Tulandi T, Koren G, Casper R (2007). Fetal safety of letrozole and clomiphene citrate for ovulation induction. J Obstet Gynaecol Can.

